# Examining the Interaction Between Medical Information Seeking Online and Understanding: Exploratory Study

**DOI:** 10.2196/13240

**Published:** 2019-09-24

**Authors:** Rei Kobayashi, Masato Ishizaki

**Affiliations:** 1 Interfaculty Initiative in Information Studies The University of Tokyo Tokyo Japan

**Keywords:** consumer health informatics, information-seeking behavior, internet access, health communication, health literacy

## Abstract

**Background:**

Online information seeking on medical topics by patients can have beneficial effects by helping them decide on treatment options and fostering better relationships with doctors. The quality of websites and processes of seeking information online have mostly been studied, with a focus on the accuracy and reliability of websites; however, few studies have examined the relationship between other aspects of quality and the processes of seeking medical information online.

**Objective:**

This exploratory study aimed to shed light on the quality of websites used for information seeking from the perspective of understanding medical information in combination with seeking it online.

**Methods:**

The study participants were 15 Japanese university students with no problem using the internet. A questionnaire survey about health literacy (47 items on a 4-point Likert scale) and information navigation skills on the internet (8 items on a 5-point Likert scale) was conducted before participants engaged in online information seeking and qualitative interviews. The students searched for information on a disease and its treatment. The websites viewed were gathered from search behavior recorded by software and browser logs. Follow-up interviews were conducted to elicit explanations from the participants about the assignments and their views of online information seeking. The explanations were evaluated by 55 health care professionals on a 3-point Likert scale and then assessed based on their comments and the participant interviews.

**Results:**

The mean age of the participants was 20.6 years (median 21; SD 1.06). All participants were able to access reliable websites with information relevant to the assignments. The mean ratings of the students’ explanations were 108.6 (median 109; range=83-134) for the disease and 105.6 (median 104; range=87-117) for its treatment. The inter-rater reliability were 0.84 (95% CI 0.77-0.90) and 0.95 (95% CI 0.93-0.97), indicating good and excellent, respectively. The mean of the sum of the health literacy skills was 115.1 (median 115; range=80-166) and the mean for information navigation skills was 25.9 (median 26; range=17-36), respectively. Health literacy and information navigation skills were moderately correlated (*r*=0.54; 95% CI 0.033-0.822; *P*=.04). Among the four stages of health literacy, understanding and appraising (*r*=0.53; 95% CI 0.025-0.820; *P*=.04) were moderately correlated with information navigation skills (*r*=0.52; 95% CI 0.013-0.816; *P*=.046). The participants had no difficulties operating and browsing the internet and considered medical and public institution websites to be reliable; however, due to unfamiliarity with medical terms, they had difficulties choosing a site from the results obtained and comparing and synthesizing information provided by different sites. They also looked for sites providing orderly information in plain language but provided explanations from sites that gave inadequate interpretations of information.

**Conclusions:**

This study revealed interactions between searching the internet for, and understanding, medical information by analyzing the processes of information seeking online, physicians’ evaluations and comments about the participants’ explanations, and the participants’ perceptions.

## Introduction

As information becomes increasingly prevalent in modern society, patients can now gather medical and health-related information through different media, enabling them to more easily find doctors and understand treatments, which in turn affects how they interact with health care professionals [1,2]. In a review by Tan and Goonawardene, it was noticed that seeking information online can improve the doctor-patient relationship [3]. It has also been noted that “Dr Google,” which refers to seeking health and medical-related information on Google, can strengthen the relationship between information seekers and health care professionals [4]. In Japan, the internet has been ranked by cancer patients as the second most trustworthy source of information after health care professionals, indicating that a large amount of information is being gathered online and that searching for it may improve patients’ understanding of their disease, thus fostering relationships with health care professionals [5]. It has also been suggested that online information seeking may reduce the prevalence of delayed diagnoses [6].

Health and medical information on the internet have been studied from the perspectives of their accuracy, their reliability, and in terms of information seeking processes. However, the accuracy of online information is often questioned [7-11], and there are concerns that searches for online health information increase patient anxiety [12]. It has been reported that medical websites focus mainly on the quality of accuracy, not on more indirect indicators such as reliability, the provision of context, the qualifications of the authors, and the use or acceptance of information by consumers [13]. Concerning the reliability of websites, the Journal of the American Medical Association (JAMA) and the DISCERN guidelines recommend that websites should display items such as the authors, affiliations, disclosures, and currency to facilitate users’ retrieval of credible information [7,14,15]. The Health On the Net (HON) code shows that websites provide useful and reliable health and medical information online [16]. The National Institutes of Health (NIH) have provided a checklist for judging the reliability of websites based on whether the sponsor or owner of the site is a Federal agency, medical school, or large professional or nonprofit organization, is related to one of those, or if not, is sponsored by such organizations, written by a health care professional, or references trustworthy sources for its health information [17]. As an example of a health information website, MedlinePlus is well known to offer reliable information on over 1000 health-related topics [18].

In addition, the actual processes of users’ information seeking for medical information have been explored qualitatively [19-23]. These studies examined how users search the internet to find answers to given assignments. Observations with in-depth interviews showed that adults in Germany could find health information to answer questions, but their search techniques were suboptimal [19]. Around 70% of the adolescent participants taking part in a study in the United States could find correct and useful answers to health questions [20]. Patterns of cognitive processes in medical information seeking were explored in young adults in the United States, and the results showed that dual processing (deliberate thinking) was associated with higher education levels and younger age. Health literacy has been linked to literacy and has been shown to entail:

People’s knowledge, motivation, and competence to access, understand, appraise, and apply health information in order to make judgments and take decisions in everyday life [24].

Thus, identifying problematic areas in terms of skills may be one way of enabling sound information seeking.

The relationship between health literacy and observations on medical information seeking was also investigated. Adults with rheumatic diseases taking part in a survey in the Netherlands experienced difficulties, especially in using search strategies and evaluating the relevance and reliability of websites [22]. Concerning health literacy, Israeli adults aged 50 years and older were shown to have lower successful completion rates of seeking medical information online in the order of accessing, understanding, appraising, applying, and generating new information [23].

Although these previous studies have deepened our understanding of health and medical information seeking online, the goals of consumers searching for medical information on the internet involve not only finding the information on a disease and its treatment options, but also understanding it for themselves, their family, or close friends. Therefore, the present exploratory study attempts to address the problem of interaction between understanding information closely related to the “use or acceptance of information” [13] and searching for medical information on the internet. For this purpose, we pose the following two research questions: (1) are consumers who do not have difficulty utilizing the internet able to find websites that offer reliable medical information; and (2) are consumers who find reliable websites able to understand the relevant medical information?

Additionally, to further the discussion of this study, we also examined health literacy and information navigation skills. Based on the answers to these research questions, the present study examined the problem of the interaction between searching the internet for, and understanding, medical information, and it suggests a future direction for effective information seeking online.

## Methods

### Participants

The study participants were recruited through a research company in Japan. The company selected a similar ratio of male and female university students from its monitors, excluding those who were majoring in medicine. To avoid any problems associated with internet use and basic reading or writing ability, all the participants were chosen from among university students, and all the students confirmed they had no problems using the internet in everyday life. The number of participants was chosen in reference to existing studies [19,20] and the recommended rule of thumb for interview surveys [25].

Written informed consent was obtained at the beginning of the study. Personal information capable of identifying individuals was kept secure by the research company.

### Study Design

#### Overview

The study was conducted in September 2017 and consisted of three stages: (1) assessing the participants’ health literacy and information navigation skills on the internet; (2) observing the participants’ information seeking behavior for given assignments; and (3) conducting follow-up interviews with the participants, and then rating and commenting on their explanations with a group of physicians. To exclude any influence from the search histories of other users, separate accounts were created for each participant. Stage 2 of the research was meant to answer for answering RQ1, stage 3 was meant to answer RQ2, the discussion of which is deepened with the results of stage 1.

#### Stage 1

The health literacy and information navigation skills of the participants were surveyed using the translated Japanese version of the Health Literacy Scale (HLS-EU-Q47), which is composed of 47 items rated on a 4-point Likert scale from 1-4 (very easy to very difficult; inverted scale) along with 0 (don’t know) [26,27], and the Information Navigation Skills on the Internet Scale [28], which is composed of eight items rated on a 5-point Likert scale from 1-5 (not at all true of me to very true of me; inverted scale).

#### Stage 2

To observe the participants’ information seeking behavior, they were allocated the assignments and instructed to search the internet for a maximum of 20 minutes. Eysenbach et al’s search experiments took 5 min 42 secs (median 4 min 18 secs; range=38 secs-20 min) per question to find an answer [19]. Hansen et al’s experiments took an average of 5 min and 41 secs and from just under a minute to nearly 24 min [20]. Perez et al’s experiments took 5 minutes 8 seconds (range=55 secs-14 min 16 secs) [21]. Based on these studies, we chose 20 minutes for the assignments, expecting that this would leave ample time for the participants. The task was to explain, in an easy to understand manner to an individual with no medical knowledge, the histological types of lung cancer (nonsmall cell lung cancer), disease staging (T2a, N1, and Stage IIB), and treatment options. In Japan, smoking is legally permitted for adults over 20 years of age. Although around 50% of high school students enter universities, they are often placed in a position of deciding whether to start smoking. As the World Health Organization has run anti-smoking campaigns regarding the risk of lung cancer, and the Olympic Games are planned for Tokyo in 2020, this anti-smoking campaign has been seen widely in Japan, giving Japanese university students opportunities to think deeply about smoking. The research team recorded information seeking processes by documenting search histories using the browser log function and screen recording software (Apower Screen Recorder Pro 2.2.4, Hong Kong). The participants bookmarked the necessary websites and took notes while seeking information.

#### Stage 3

The follow-up interviews were conducted immediately after the online search for the given assignments. An interview guide that had been prepared in advance was used to ask the participants to explain the disease and its treatment options and how they perceived the online information seeking. The interviews were recorded using a digital voice recorder and then transcribed verbatim. A consumer’s ability to understand information differs from that of external observers; thus, in this study, the participants explained their answers using the websites they had bookmarked and the notes they had taken while performing the search. A total of 55 thoracic surgeons rated the participants’ explanations on a 3-point Likert scale, from 1 (correct) to 3 (incorrect), and then provided comments. Low-rated explanations by the participants who were able to visit the websites that had adequate information were examined based on the physicians’ comments and the interviews.

All statistical computations were performed using R version 3.5.2 (The R Foundation, Vienna, Austria). This study was approved by the Institutional Review Board of the Interfaculty Initiative in Information Studies, The University of Tokyo.

## Results

### Participants

The participants consisted of seven females and eight males (mean age 20.6; median age 21; SD 1.06).

### Reliability of Websites

Table 1 summarizes the websites visited by the participants, their staying time as extracted from the logs of the browser with the captured screen records, and their ratios. The websites were classified by the first and the second authors using the scheme proposed in Goto et al [7], and all differences were resolved by discussion. The classification consists of nonprofit organizations and public institutions (PI), medical institutions (MI), pharmaceutical companies (PC), commercial companies (CC), medical professionals (MP), encyclopedias or dictionaries (ED), and unknown. Analysis of the search results using the words that appeared in the assignments revealed that the participants visited a mean of 5.9 sites (median 6; range=3-10), and their mean staying time was 177.6 secs (median 105.1 secs; range 2.33-918.4). All participants were confirmed to have reached websites matching a checklist issued by the NIH [17] for judging reliable websites, such as those sponsored or owned by a federal agency, large PI or MI, or written by a PC referencing trustworthy sources for its health information.

**Table 1:** The websites visited by the participants (P1-P15), their staying time (in seconds), and their ratios (in brackets; calculated as the ratios of staying time for respective sites divided by that for all sites).

Websites	P1^a^	P2	P3	P4	P5	P6	P7	P8	P9	P10	P11	P12	P13	P14	P15
PI^b^ 1	—^c^	187.32 (20.3)	61.79 (5.9)	412.33 (36.9)	164.96 (14.0)	—	252.35 (23.7)	—	73.74 (6.7)	—	483.35 (58.8)	118.25 (10.7)	168.56 (14.8)	—	—
PI 2	86.25 (9.5)	—	—	—	—	—	—	—	289.34 (26.5)	192.95 (16.5)	—	237.53 (21.5)	793.74 (69.7)	—	269.25 (29.2)
PI 3	—	11.61 (1.3)	—	—	—	—	—	—	5.24 (0.5)	—	—	27.33 (2.5)	—	154.97 (13.2)	3.74 (0.4)
PI 4	—	55.57 (6.0)	—	—	—	—	—	—	—	—	—	—	—	—	—
PI 5	—	—	—	—	—	—	—	115.92 (10.0)	—	—	—	—	—	—	—
MI^d^ 1	87.25 (9.6)	36.38 (3.9)	151.64 (14.4)	104.58 (9.4)	451.58 (38.3)	232.66 (23.0)	—	—	73.67 (6.7)	56.93 (4.9)	21.97 (2.7)	22.66 (2.0)	—	—	—
MI 2	—	78.84 (8.5)	—	16.92 (1.5)	423.89 (36.0)	—	—	—	24.51 (2.2)	—	—	—	—	66.05 (5.6)	105.51 (11.5)
MI 3	—	211.45 (22.9)	—	—	—	—	—	—	—	—	—	—	36.38 (3.2)	—	—
MI 4	—	—	133.27 (12.6)	—	—	—	—	—	—	—	—	—	—	—	—
MI 5	—	—	290.27 (27.5)	—	—	—	—	—	—	—	—	—	—	—	—
MI 6	—	—	—	—	—	337.91 (33.4)	—	—	—	—	—	—	—	—	—
MI 7	—	—	—	—	—	188.34 (18.6)	—	—	—	—	—	—	—	—	—
MI 8	—	—	—	—	—	—	—	—	—	—	—	261.49 (23.7)	—	—	—
PC^e^ 1	—	306.39 (33.2)	379.49 (36.0)	438.81 (39.3)	139.05 (11.8)	251.62 (24.9)	743.11 (69.9)	903.68 (77.9)	262.3 (24.0)	918.43 (78.6)	192.64 (23.4)	330.55 (30.0)	140.86 (12.4)	215.22 (18.3)	518.05 (56.3)
PC 2	—	9.26 (1.0)	16.07 (1.5)	72.58 (6.5)	—	—	—	—	—	—	—	14.74 (1.3)	—	—	13.77 (1.5)
PC 3	—	—	—	—	—	—	31.33 (3.0)	–	67.23 (6.1)	—	88.15 (10.7)	—	—	70.33 (6.0)	—
PC 4	—	—	—	—	—	—	—	—	—	—	36.34 (4.4)	—	—	–	—
CC^f^ 1	346.48 (38.0)	—	—	—	—	—	—	—	164.78 (15.1)	—	—	93.05 (8.4)	—	127.42 (10.8)	—
CC 2	2.33 (0.3)	—	—	—	—	—	—	—	—	—	—	—	—	—	—
CC 3	—	18.09 (2.0)	—	—	—	—	—	—	—	—	—	—	—	—	—
CC 4	—	–	22.63 (2.1)	—	—	—	—	—	—	—	—	—	—	—	—
CC 5	—	—	—	—	—	—	—	—	25.19 (2.3)	—	—	—	—	—	—
CC 6	—	—	—	—	—	—	—	—	—	—	—	—	—	541.93 (46.0)	—
CC 7	—	—	—	—	—	—	—	—	—	—	—	—	—	—	9.49 (1.0)
CC 8	—	—	—	—	—	—	—	—	107.29 (9.8)	—	—	—	—	—	—
MP^g^	—	—	—	—	—	—	—	48.12 (4.1)	—	—	—	—	—	—	—
ED^h^ 1	44.01 (4.8)	—	—	—	—	—	36.53 (3.4)	—	—	—	—	—	—	—	—
ED 2	—	8.35 (1.0)	—	—	—	—	—	—	—	—	—	—	—	—	—
ED 3	—	—	—	72.66 (6.5)	—	—	—	—	—	—	—	—	—	—	—
Unknown	61.24 (6.7)	—	—	—	—	—	—	92.88 (8.0)	—	—	—	—	—	—	—

^a^P: participant

^b^PI: nonprofit organizations and public institutions

^c^Not applicable.

^d^MI: medical institutions

^e^PC: pharmaceutical companies

^f^CC: commercial companies

^g^MP: medical professionals

^h^ED: encyclopedias or dictionaries

The NIH checklist is not a detailed specification of reliable sites but a set of heuristic rules for consumers when searching for reliable medical and health information on the internet. To further check whether the websites contained sufficient information for the task, the websites that the participants visited were examined for information on diseases (nonsmall cell lung cancer, T-factor, N-factor, and staging) and treatment options (surgery, chemotherapy, and radiation). Table 2 shows that the websites recommended by the NIH did not necessarily include relevant information. However, the websites participants stayed on for the longest to second-longest and the longest to the third-longest time had requisite information for the disease and its treatment, respectively.

### Understanding Relevant Medical Information

#### Ratings of the Explanations by Medical Professionals

##### Overview

The participants were interviewed after searching the internet for the given assignments, and during the interviews they explained the disease and its treatment and described their perceptions of the internet search. After that, 55 thoracic surgeons rated the correctness of the participants’ explanations on a 3-point Likert scale from one (correct) to three (incorrect) and provided comments regarding the explanations. Their mean ratings were 108.6 (median 109; range=83-134; min-max [refers to theoretical range]=55-165) for the disease and 105.6 (median 104; range=87-117; min-max=55-165) for its treatment options. However, the judgments of the MP were different from coders’ ratings based on a coding book. To assess the reliability of the rating, the inter-rater reliability, ICC(3,k), which is frequently utilized in computing internal consistency [29], was calculated. The reasons that the ICC(3,k) were chosen are: (1) the same set of raters are used for all subjects; (2) it is based on mean of the raters; and (3) for consistency, it is more appropriate than absolute agreement for the judgments of the MP. The results obtained were 0.84 (95% CI 0.77-0.90), indicating good, and 0.95 (95% CI 0.93-0.97), indicating excellent.

Below, low-rated participants (ie, participants who scored below average) were considered to not have any understanding of the medical information. Difficulties they faced were examined based on their interviews and the physicians’ comments on their responses.

##### Explanations for the Disease

Overall, 8 participants whose explanations were rated below average were able to access websites that had information on the disease, but they were unable to extract enough information to answer the question. In the interviews, 5/8 participants expressed difficulties in understanding unfamiliar technical terms. The physicians provided comments that their explanations included inadequate information extracted from inappropriate locations on the websites and given as incorrect answers.

**Table 2:** The websites visited by the participants (P1-P15) and whether they included information about the disease and its treatment.

Websites	Disease								Treatment options		
	Non-small cell lung cancer	T-factor	T2a	N-factor	N1	Stage	Stage ⅡB	Surgery		Chemotherapy followed by surgery	Radiation therapy for patients who cannot have surgery
PI^a^ 1	✔	✔	✔	✔	✔	✔	✔	✔		✔	✔
PI 2	✔										
PI 3			✔	✔	✔	✔	✔	✔		✔	✔
PI 4											
PI 5	✔	✔		✔		✔		✔		✔	✔
MI^b^ 1	✔	✔	✔	✔	✔	✔	✔	✔			✔
MI 2	✔					✔		✔		✔	✔
MI 3	✔		✔	✔	✔		✔	✔		✔	✔
MI 4	✔	✔	✔	✔				✔		✔	
MI 5	✔	✔	✔	✔	✔	✔	✔	✔		✔	✔
MI 6	✔			✔			✔	✔		✔	✔
MI 7	✔	✔	✔	✔	✔	✔	✔	✔		✔	✔
MI 8	✔		✔	✔	✔		✔	✔			✔
PC^c^ 1	✔	✔	✔	✔	✔	✔	✔	✔		✔	
PC 2								✔		✔	✔
PC 3	✔					✔		✔		✔	
PC 4	✔										
CC^d^ 1	✔							✔		✔	✔
CC 2						✔		✔			
CC 3	✔										
CC 4											
CC 5											
CC 6	✔			✔		✔		✔		✔	✔
CC 7	✔			✔	✔	✔		✔		✔	
CC 8						✔					
MP^d^											
ED^f^ 1	✔		✔	✔		✔					
ED 2	✔		✔	✔	✔	✔	✔	✔			
ED 3	✔					✔		✔		✔	
Un-known		✔		✔	✔			✔		✔	

^a^PI: nonprofit organizations and public institutions

^b^MI: medical institutions

^c^PC: pharmaceutical companies

^d^CC: commercial companies

^e^MP: medical professionals

^f^ED: encyclopedias or dictionaries

##### Explanations for the Treatment Options

In total, 7 participants whose explanations were rated below average were able to access websites that had information about treatment options. However, regardless of rating, none of the participants were able to extract enough information to answer the question. Some of the websites did not include enough information for all treatment options, so the participants who visited such websites needed to synthesize information from multiple sites, which made the task more demanding. The physicians commented that the responses provided by 5/7 participants who were rated below average included either incorrect expressions or inadequate information extracted from inappropriate locations on the websites they used.

##### Participants’ Health Literacy and Information Navigation Skills

The mean, median, min-max, and range of the sum of the health literacy skill scores were 115.1, 115, 47-188, and 80-166, respectively, which are comparable to the results of Nakayama et al’s study involving the Japanese population [27], and the mean, median, min-max, and range of the sum of the information navigation skill scores were 25.9, 26, 8-40, and 17-36, respectively. The health literacy and information navigation skills were moderately correlated (r=0.54; 95% CI 0.033-0.822; *P*=.04). Among the four stages of health literacy (accessing, understanding, appraising, and applying), understanding and appraising (r=0.53, 95% CI 0.025-0.820; *P*=.04) were moderately correlated with internet literacy (r=0.52; 95% CI 0.013-0.816; *P*=.046).

## Discussion

### Reliability of Websites

The participants were able to find sites that followed the NIH guidelines and included relevant information for the assignments, but each site did not necessarily have a complete collection of information.

The participants in the present study did not report having any difficulties operating the browser or searching the internet; however, their level of health literacy was a little below the theoretical average, comparable to the results of Nakayama et al’s survey on the Japanese population [27], while their level of information navigation skills was a little above the theoretical average. These results may be because of differences between self-reported and questionnaire-based health literacy. Information navigation skills on the internet were moderately correlated with understanding and appraising health literacy, which indicates that the former involves some aspect of comprehending health and medical information on the internet.

Neter and Brainin [23] showed that self-administered health literacy was moderately correlated with actual health literacy. This means that currently the former cannot be an accurate index for the latter, and thus, it is difficult to distinguish patients with low health literacy (LHL) from those with high health literacy (HHL). Furthermore, medical information for patients with LHL can be used for those with HHL, but not vice versa. Therefore, the written and online strategies reviewed by Noordman et al [30] can be used to support consumers with either LHL or HHL to select a website, compare multiple sites, and understand information on the internet, at least until more accurate health literacy–reflecting actual behavior is developed, as noted by Neter and Brainin [23].

### Understanding Relevant Medical Information

An analysis of the physicians’ ratings and comments about the explanations, as well as the interview data for the participants, revealed an interaction between information seeking online and understanding. That is, even if the participants visited the websites of a PI or MI that provided correct medical information, they would go to another site to obtain the same information explained simply, but then they would process this information inadequately. These participants did not have enough knowledge to understand medical information, they had trouble sifting through the large number of search results, and they found it difficult to compare and synthesize information from different sites to obtain answers to their medical questions.

Noordman et al reviewed several strategies and tools for health care professionals to support patients with LHL in hospital-based palliative care settings [30]. The written and online strategies were classified into those related to content (providing information in lay terminology and developing test material for the target population) and those related to representation (the use of graphs and illustrations, font size and spacing, and the length of sentences and paragraphs). They suggested that the strategies and tools were not specific to the palliative care setting for patients with LHL.

The findings of this study regarding the interaction between information seeking online and understanding medical information suggest the possibility of considering the quality of medical information from the viewpoint of understanding it, in combination with the process of information seeking. Pallotti et al’s study to integrate the readability of a website into their search ranking algorithm [31] can be considered a step in this direction.

Evaluating consumers’ understanding is not a simple task. The assessments carried out by the physicians in the present study are too costly to apply. Instead, test materials for information on a website, as suggested by Noordman et al, may be a tool for consumers to self-check whether they can grasp the level of information. Eysenbach and Diepgen proposed self-labelling of medical information by website authors with a systematic evaluation of health-related information by users and third parties using a legitimized standard vocabulary [13]. Their proposal has not been widely used; however, the test materials may be an approximate substitute for website authors’ self-labels, in that consumers are able to judge a website by reviewing these as a kind of summary instead of viewing the complete information.

### Limitations

Qualitative studies and quantitative studies are complementary: the former can examine the details of phenomena and propose assumptions consisting of novel concepts and their relationships (although these need to be generalized), whereas the latter can verify a theory based on statistics, although this sometimes involves assumptions that are not free from questions regarding the validity of statistical inferences. This dichotomy of characterization may be coarse, but it is unavoidable that both approaches are necessary to advance research.

As this was only an exploratory study, further research with more diverse participants and assignments is needed to increase the generalizability of the findings. Furthermore, those who search for medical information online sometimes engage in information seeking because of a vague sense of unease without having a clear sense of what they are searching for. In the present study, the participants had a clear sense of what they were searching for and therefore, future studies could explore the search behaviors of individuals who do not. In addition, future research should include test materials for a website prepared to examine how to assist consumers with internet searching.

## Abbreviations

CC: commercial companies
ED: encyclopedias/dictionaries
HHL: high health literacy
HLS: health literacy scale
HON: Health On the Net
ICC (3,k): intraclass correlation coefficient (3,k)
JAMA: Journal of the American Medical Association
LHL: low health literacy
MI: medical institutions
MP: medical professionals
NIH: The National Institutes of Health
PC: pharmaceutical companies
PI: nonprofit organizations and public institutions
