# Correction: Examining the Interaction Between Medical Information Seeking Online and Understanding: Exploratory Study

**DOI:** 10.2196/35222

**Published:** 2021-12-02

**Authors:** Rei Kobayashi, Masato Ishizaki

**Affiliations:** 1 Interfaculty Initiative in Information Studies The University of Tokyo Tokyo Japan

In “Examining the Interaction Between Medical Information Seeking Online and Understanding: Exploratory Study” (JMIR Cancer 2019;5(2):e13240) the authors noted one error.

In the originally published manuscript, the following sentence appeared under the “Understanding Relevant Medical Information” and “Ratings of the Explanations by Medical Professionals” section:

To assess the reliability of the rating, the inter-rater reliability, Cronbach alpha, which is frequently utilized in computing internal consistency [29], was calculated.

This sentence has been corrected to:

To assess the reliability of the rating, the inter-rater reliability, ICC(3,k), which is frequently utilized in computing internal consistency [29], was calculated.

The correction will appear in the online version of the paper on the JMIR Publications website on December 2, 2021, together with the publication of this correction notice. Because this was made after submission to PubMed, PubMed Central, and other full-text repositories, the corrected article has also been resubmitted to those repositories.

